# Urban Area Extent Extraction in Spaceborne HR and VHR Data Using Multi-Resolution Features

**DOI:** 10.3390/s141018337

**Published:** 2014-09-30

**Authors:** Gianni Cristian Iannelli, Gianni Lisini, Fabio Dell'Acqua, Raul Queiroz Feitosa, Gilson Alexandre Ostwald Pedro da Costa, Paolo Gamba

**Affiliations:** 1 Department of Industrial and Information Engineering, University of Pavia, Via Ferrata 5, Pavia 27100, Italy; E-Mails: fabio.dellacqua@unipv.com (F.D.A.); paolo.gamba@unipv.com (P.G.); 2 Institute for Advanced Studies, Palazzo della Vittoria, Pavia 27100, Italy; E-Mail: gianni.lisini@iusspavia.it; 3 Department of Electrical Engineering, Pontifical Catholic University of Rio de Janeiro, Marquês de São Vicente, 225, Gávea 22451-900, Brazil; E-Mails: raul@ele.puc-rio.br (R.Q.F.); gilson@ele.puc-rio.br (G.A.O.P.C.)

**Keywords:** urban areas, multi-resolution processing, feature fusion

## Abstract

Detection of urban area extents by means of remotely sensed data is a difficult task, especially because of the multiple, diverse definitions of what an “urban area” is. The models of urban areas listed in technical literature are based on the combination of spectral information with spatial patterns, possibly at different spatial resolutions. Starting from the same data set, “urban area” extraction may thus lead to multiple outputs. If this is done in a well-structured framework, however, this may be considered as an advantage rather than an issue. This paper proposes a novel framework for urban area extent extraction from multispectral Earth Observation (EO) data. The key is to compute and combine spectral and multi-scale spatial features. By selecting the most adequate features, and combining them with proper logical rules, the approach allows matching multiple urban area models. Experimental results for different locations in Brazil and Kenya using High-Resolution (HR) data prove the usefulness and flexibility of the framework.

## 1. Introduction

Methodologies developed to extract human settlements from remote sensing data are usually (and correctly) driven by the sensor type and the spatial/spectral resolutions. For coarse spatial resolutions, spectral/backscattering information is considered the most efficient way to discriminate between urban and rural environments, as demonstrated by papers using nighttime light data [1] or Synthetic Aperture Radar (SAR) Wide Swath Mode images [2]. When multispectral bands are available, it becomes possible to exploit spectral indexes obtained by combining reflectance values at different wavelengths in either linear [3] or non-linear ways [4]. With higher spatial resolution data, textural features are considered as relevant or even more important than spectral features [5], to the extent that some authors feel they are the most relevant (sometimes, the sole) features to be considered [6].

Additionally, the problem of individuating which “urban area parts” are captured by remotely sensed data has been consistently one of the main disputed points in global urban remote sensing research. Once again, opinions are driven by the spatial and spectral resolution of the used EO data. “Impervious surfaces” and “city extents” are favored with multispectral and coarser resolution data sets. “Built-up structures” and “settlement structural features” are the targets of Very High Resolution (VHR) and panchromatic image analysis algorithms.

Finally, it has been remarked [7] that the analysis of urban remote sensing imagery is inherently multi-scale, because in urban areas multiple features of different sizes are spatially, logically, or semantically connected. Footprints of meaningful objects may be inferred from their parts. Conversely, coarser resolution elements may help in focusing attention on finer details. Similarly, recent works about High Resolution (HR) images [8–12] have observed that, even in other mapping applications, better accuracies can be achieved by exploiting multi-scale feature extraction techniques.

Therefore, the combination of multiple features at different spatial resolution is apparently an effective way to exploit the information available in Earth Observation (EO) data for urban area analysis. Accordingly, this paper proposes a novel flexible framework for automatic urban area delineation. With respect to other approaches reported in literature, the contributions of this work are three-fold, and largely improve our previously presented methodology [13].

- The framework is able to accommodate for a variety of “urban area” definitions;
- Additionally, it provides a flexible and powerful set of tools for describing/modeling images of urban areas;
- Finally, it allows the exploitation of multisensor and multiresolution remotely sensed data.

The paper is structured as follows: Section 2 provides a brief summary of the most relevant technical works on the subject of urban area extent extraction, with a focus on different “urban area” definitions, while Section 3 introduces the proposed framework and discusses in details its steps. Section 4 is devoted to the experimental results obtained on HR and VHR data from different geographical locations. Finally, Section 5 highlights the qualifying points of this work, and discusses its improvements with respect to the state-of-the-art without hiding its limitations.

## 2. Related Work on Urban Area Extraction

As mentioned in the Introduction, there have been several attempts to design methods and extract “urban areas” from EO data. However, these methods were always tailored for one specific definition.

Specifically, the approach followed in [14] for the MODIS500 data set, currently the most widely used global urban layer, aims at extracting urban areas according to an “urban land use” model. Moreover, due to the rather coarse spatial resolution of the input data, this approach does not consider small towns and villages, and was validated by means of a very large test set, but composed predominately of large cities.

The algorithm described in [15,16], on the contrary, sees human settlements as agglomerates of buildings and other artificial structures (*i.e.*, “built-up areas”). Therefore, it requires VHR data as input, and employs a bottom-up technique. It starts from the extraction of built-up structures. Then, built-up density is obtained. Finally, by considering spatial aggregations at different resolutions, different urban area extents are extracted. Similarly, in [17], Gabor texture filters are used to extract salient points in built-up areas, subsequently combined to select building agglomerates.

Landsat data are also often used for urban area extraction. They have an intermediate resolution between the two previous examples, but a huge legacy of multitemporal acquisitions. The published papers show however a large variability of urban area models. In [18], for instance, multitemporal spectral information is used to extract “places dominated by the built environment”, defined as “… all non-vegetative, human-constructed elements, such as roads, buildings, runways.” [19]. Therefore, citing from the same paper, parks, and water ponds are not considered parts of the urban area, “… although they may function as urban space.” In [20], instead, a totally different urban area definition is used, including urban parks and looking for compact and convex boundaries. This approach was applied to monitor the development of 663 Chinese cities from 1990 to 2010. Moreover, in [21], a novel multiple index, the Normalized Spectral Difference Vector, is used to discriminate on a pixel-per-pixel basis urban and artificial materials from natural ones, essentially looking for “urban land cover” extents. Finally, dictionary-based recognition algorithms are applied to VHR but also to coarser resolution data sets extract urban extents according to sample urban patches [22].

## 3. The Proposed Framework

As already mentioned, the framework proposed in this work involves the extraction of spectral and spatial features at multiple spatial resolutions, eventually combined to extract “urban areas”.

Indeed, it may be expected that most “urban area” models can be represented by a combination of the clues described in the following paragraphs.

- *Density of built-up structures*: from an ontological point of view, generic human settlements are labeled “urban areas” depending on the density of the artificial structures. Accordingly, density of artificial structures can be used to distinguish between urban and rural areas. It may also be exploited to discriminate portions of urban areas, like dense industrial districts from sparse residential areas. In remotely sensed data, textural features can be used to capture density (e.g., less dense areas are also low-contrast areas). Additionally, different urban densities may result into different average reflectance values in each urban block. Accordingly, both spectral properties and spatial patterns can be used as image descriptors related to built-up structure density.
- *Number of built-up structures*: in addition to built-up structure density, absolute building numbers are also important. As a matter of fact, the difference between isolated farms, small villages and big cities is not only due to their density, but also to the number of structures that lie together in close patterns. In remotely sensed data, the absolute number of buildings can be inferred from a building detection step. Note that, under the assumption that missed buildings are uniformly distributed all around the scene, even if this number is not very accurate, the relevance of the produced information is still maintained.
- *Urban vegetation/water*: urban vegetation and water bodies are sometimes considered as parts of an urban area, e.g., for environmental applications. The same areas are not considered however as a part of the urban area for some other applications, such as for exposure (which commonly refers to built-up structures only), or for the analysis of impervious surfaces. In remotely sensed data, hints of vegetation and water can be obtained by means of spectral indexes, such as the Normalized Difference Vegetation Index (NDVI). According to the adopted definition of urban area, however, this spectral information can be useful or may be totally irrelevant.

According to this overview, a flexible processing framework to characterize “urban areas” from remotely sensed data must be able to provide quantitative information, or at least hints, of all of these clues. Eventually, this information will be combined to extract the “urban area” of interest to the user. The processing chain implemented in this work is therefore structured into four steps, shortly summarized here and detailed in the following sections.

- *Extraction of built-up areas*: the first step delineates at a coarse scale the portion of the scene that may be related to urban areas. According to current state-of-the-art (e.g., [23] or [24]) this area corresponds to built-up structures and their surrounding environment. This step is carried out at spatial resolutions between 5 and 10 m, corresponding to the usual size of small buildings. If the data spatial resolution is finer than 5 m, it is recommended to resample the data before executing this step.
- *Built-up structure proxy extraction*: the full spatial resolution of the data is then used to extract hints of buildings, or single built-up structures. This is done by binary unsupervised classification (with or without segmentation) of spatial and/or spectral image features.
- *Spectral feature extraction*: in order to include information about natural and artificial materials (e.g., vegetation and water bodies), pixel-based spectral indexes (e.g., the already mentioned NDVI), and average spectral reflectance values are computed for each candidate area.
- *Feature combination*: the final step combines the spectral and multi-scale spatial features using logical AND/OR operators. Please note that we refer to multi-scale spatial features because we consider both the extraction at coarse resolution and at full resolution (the first and second step, respectively).

**Figure 1.**
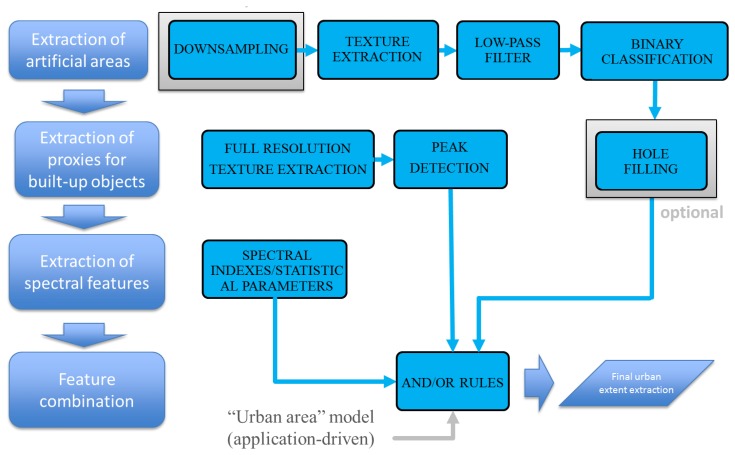
Graphical representation of the overall processing framework proposed in this paper. The framework includes spectral and multi-scale spatial features, independently extracted from the input data and eventually combined according to logical rules (AND, OR) to match the urban area definition most suitable to the final application needs.

A schematic representation of the approach is illustrated in Figure 1.

### 3.1. Artificial Area Extent Extraction

To provide a coarse-resolution approximation of “urban areas” the first processing step aims at delineating areas in an image potentially enclosing human settlements. This step is implemented by the Urban Focus (UF) software tool developed in the framework of the BREC suite [25]. UF computes a generalized version of the PanTeX index [6]. UF detects built-up areas, under the assumption (validated *a posteriori* by the extraction results, see again [25]) that buildings and other built-up artificial structures show more contrast than many natural environments. Moreover, UF computes and combines the contrast among multiple directions with a window size in the order of 100 m × 100 m. At this scale, directionality due to the road network is discarded, and only the high contrast due to built-up structures is captured.

Please note that other textures may have been used to the same aim: the Grey-Level Co-occurrence Matrix (GLCM) [26] or the Local Index of Spatial Association (LISA) features [27], as well as morphological operators [28].

**Figure 2.**
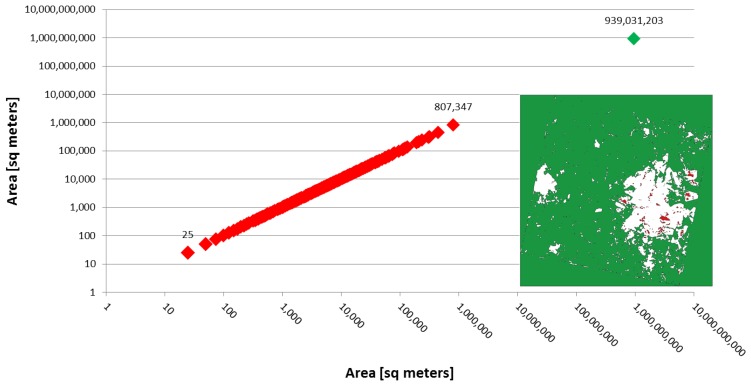
Distribution of area values for non-built-up areas (in square meters) for the figure on the right (white = urban area, red = “holes”, green = natural background). Note the log scale on both axes.

Once the texture feature is extracted, the results are processed by means of a low-pass filter with kernel size comparable to an urban block. This step smoothens the extracted feature in the range of 100 m. The effects are the reduction of isolated false positives caused by small pixel agglomerates, and the extraction of more homogeneous areas. Finally, a “hole filling” technique is applied. Specifically, some instances of built-up regions in the image may contain “holes” (e.g., parks, vegetation, water bodies, *etc.*). These “holes” (as well as the natural parts of the scene) are represented by connected components within and around the built-up regions. The “holes” are usually much smaller than the other natural part of the scene. Hence, they can be easily recognized by a threshold on their area, and eventually labeled as built-up. The final result is, thus, a mask of built-up regions without small holes. We stress that the choice of the area threshold value is not critical, as the areas of such holes are concentrated in a narrow range far below the area of the other natural portions of the scene (see Figure 2).

### 3.2. Built-Up Structure Proxy Extraction

Essentially the same procedure followed in the first step is adopted here with three differences. First, not subsampling, nor low-pass filtering, are applied. Second, small, connected components are not removed. Third, a higher threshold is applied to the UF index values, in order to detect only its peaks. In this way, structures at the finest available resolution are enhanced and properly delineated.

For small scenes it is also possible to rely on a segmentation of the area. After segmentation, different features can be considered to detect hints of buildings, such as the area, shape, length, square-ness, brightness, and so on [29]. These parameters must be, however, tuned to the scene. This is the reason why in this paper the first method is used, resulting into a simpler and faster algorithm, with just one parameter (a threshold) to be set by the user.

### 3.3. Spectral Feature Extraction

Spectral features are very relevant either to characterize the different parts of the urban environment, or to discriminate artificial and natural materials, or finally to detect natural elements within the urban boundaries. This information is extracted by means of pixel-based indexes, but also by considering statistical parameters, like the mean reflectance or standard deviation for the spectral bands of interest. In this sense, spectral information may be used at the pixel level, but also at the segment level, whatever the spatial scale (from a single block to the whole town).

### 3.4. Feature Combination

The last part of the processing chain combines the outputs of the previous steps and refines the result according to the “urban area” model of interest to the user. Specifically, in our framework, the urban area model is matched by simple operations, like mask and/or intersect applied to different extracted features at different spatial scales. The existence (and characteristics) of a settlement depends on a combination of those features. In our approach this “fusion” step is implemented by a set of rules based on logical “AND” and “OR” operations. This method has the advantage, in regard to other methodologies, of being compatible to multiple urban area definitions. In other words, while most existing methods have been designed considering just one model of urban area, in this research the method is designed thinking—in principle—of any possible model of urban areas. Moreover, these models can be matched by asking the user to describe them in terms of few simple intuitive features related to the ontology of an urban area. As a consequence, the proposed method can be adjusted to different urban typologies by users without a strong knowledge of remote sensing image interpretation.

### 3.5. Procedure Parameters

According to the discussion above, there are three types of parameters within the proposed framework:

- Automatic: parameters already set, with no need for tuning; examples are the resolution to which data is sub-sampled for UF, as well as the window size used for its computation, automatically selected according to the block size and the spatial resolution of the data;
- Semi-automatic: parameters whose values are set by default, but which can be optimized according to the particular human settlement model to achieve more accurate results. Specifically, this is the case for the two thresholds to extract proxies to urban extents and buildings presence from textural indexes;
- Model dependent: parameters that strictly depend on the human settlement model, and must be selected on a case-by-case basis. Examples are the number of building in each human settlement, or the NDVI range of interest.

In the following section, the values of the relevant parameters for each example and test site are reported.

## 4. Experimental Results

The experimental results were obtained using HR and VHR data from different geographical locations and considering different definitions of “urban area” or “human settlement”. As described in the following paragraphs, the results are very promising and prove that the proposed framework allows analyzing different urban environments and performing different interpretation tasks in urban remote sensing.

### 4.1. Test Sites

The first test site is a region in Sao Paulo State, Brazil. The dataset used in this research was acquired by the satellite CBERS-2B [30]. This satellite carried three sensors, named High Resolution CCD Camera (HRCC), High Resolution Camera (HRC), and Wide Field Imager (WFI). HRCC is an instrument with five bands (a panchromatic image, plus RGB and NIR bands) with a spatial resolution of 20 m and a swath of 113 km. HRC is composed of just a panchromatic sensor with a spatial resolution of 2.36 m and a swath width of 27 km. Finally, the WFI acquires only two bands, Red and NIR, with a spatial resolution of 260 m and a swath width of 885 km. In this paper, we used images acquired in 2008 by the HRC sensor over the towns of Ribeirao Preto and Aracatuba, on 30 July and 10 October, respectively. The scenes were selected because of they represent different urban areas and contains settlements from very small to moderate size. The images geometrically corrected using a set of Ground Control Points, manually selected and uniformly distributed over the image. A first-order polynomial transformation method was adopted to achieve georeferentiation at the sub-pixel level.

**Figure 3.**
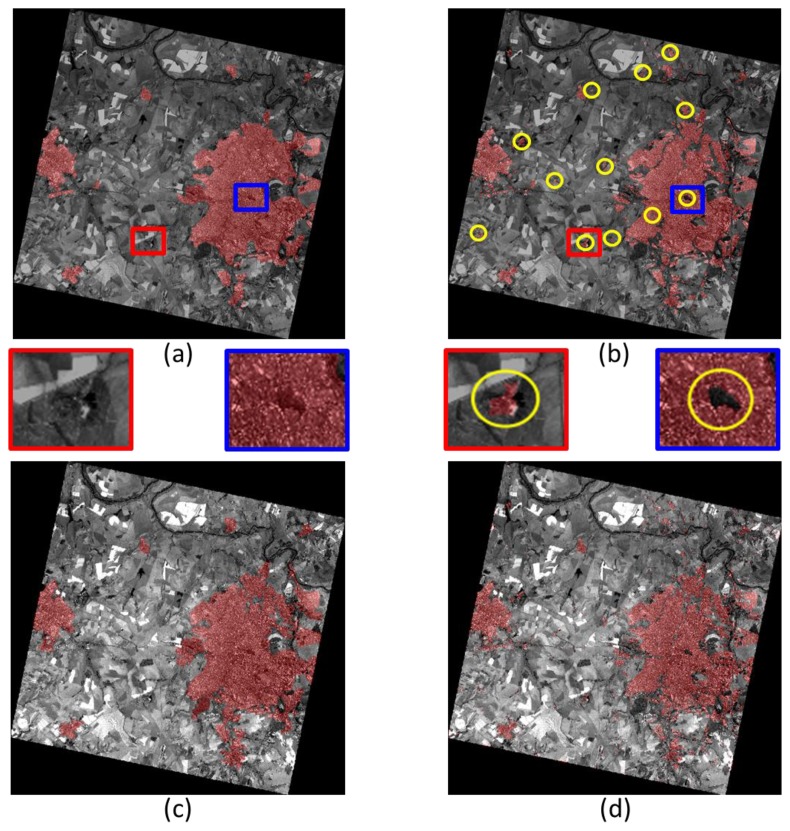
Ribeirao Preto test site: (**a**) first reference data set superimposed to the original CBERS-2B scene; (**b**) second reference data set; (**c**) results of the proposed procedure matching the first reference data set; (**d**) results of the proposed procedure matching the second reference data set.

For each of the two analyzed scenes, two different reference datasets were used for evaluation, representing two different urban area definitions: the first one (see Figure 3a for Ribeirao Preto) was produced by photo interpretation of 2004 Landsat data, updated using additional data from the same sensor in 2009 [31,32]. Since the rather coarse spatial resolution data did not permit the detection of small settlements, the extracted urban extents correspond to a Minimum Mapping Unit of 300,000 m^2^. Small settlements and isolated built-up structures (e.g., farm houses) are neglected. The second reference data sets (see Figure 3b, again for Ribeirao Preto) was obtained by visual interpretation of the CBERS images and identifies only built-up areas—with no MMU limitation—at the finest available spatial resolution. The definition underlying the first reference data includes therefore only human settlements larger than a certain size, and does not discriminate urban parks and vegetation from built-up areas. The second one, instead, stems from a definition of “urban area” where only buildings are considered, even within very small villages and including isolated farms, without considering urban water ponds/lakes and parks. Yellow circles in Figure 3b identify major differences between the two definitions as visible in Figure 3a,b. A couple of these differences have been enlarged to make this point clearer.

Finally, and in order to test the proposed framework on a very different environment and for a completely different “urban area” extraction problem, another test was run on the capital of Kenya, Nairobi. The data set used in this test is a Quickbird image, recorded on 11 February 2002. The scene is composed of a panchromatic band at sub-meter resolution and a multispectral acquisition at 2.4-meter spatial resolution. The goal of this second test is not to delineate built-up areas, but to extract informal settlements. The extraction results can be compared to slum locations and extents in Nairobi provided by the United Nations Human Settlements Programme, UN-Habitat.

**Figure 4.**
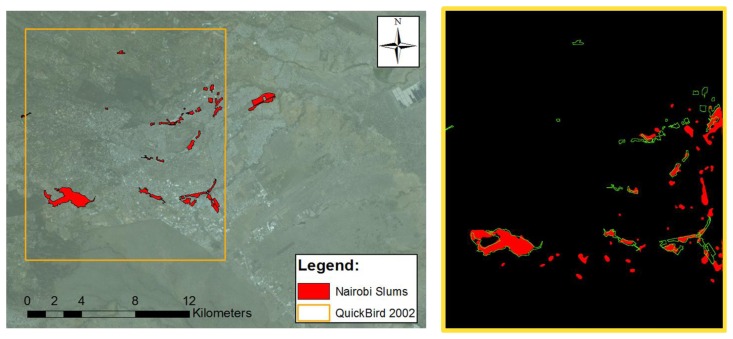
Informal settlement extraction. On the left, the area of Nairobi (Kenya) covered by the 2002 Quickbird image used in this work, with the slum extents provided by UN-Habitat superimposed in green; on the right, in red the overall slum extents extracted by the proposed algorithm compared with the boundaries of the ground truth, still in green.

The satellite image, with the UN-Habitat slum extents superimposed in green, is shown in Figure 4.

### 4.2. Extraction of “Compact Urban Areas”

**Figure 5.**
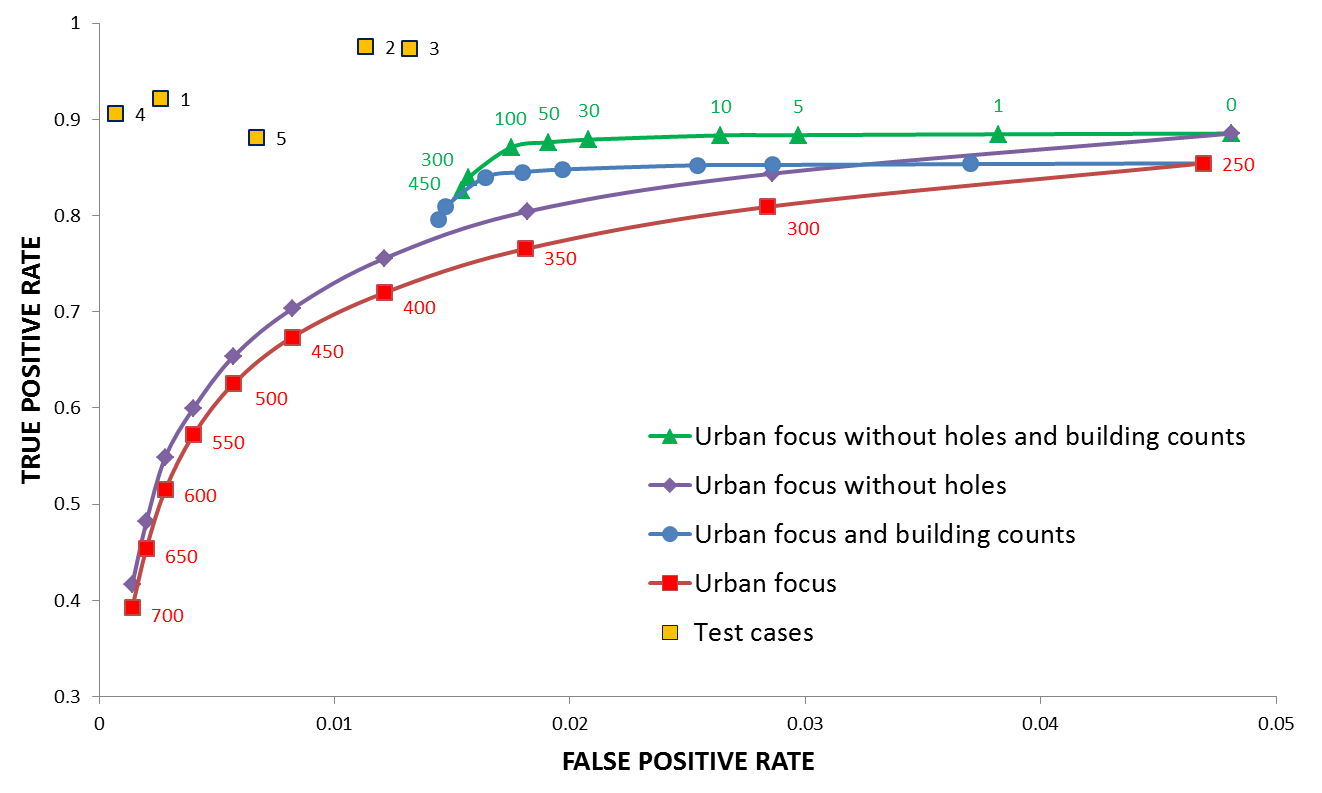
Receiver Operator Curve for the Ribeirao Preto test site against the first reference data set in Figure 3a. The term “without holes” means that the urban vegetation (e.g., urban parks) is considered as part of the urban area. The term “count” refers to the minimum number of buildings per agglomerate. Final results for five other CBERS scenes (orange squares) are also included for comparison.

A quantitative analysis of the results for Ribeirao Preto against Figure 3a (labeled here “compact urban area”) as a function of the UF threshold is graphically provided in Figure 5, using classical Receiver Operating Characteristic (ROC) curves. The red and purple curves show that the True Positive Rate (TPR), *i.e.*, the percentage of pixels correctly matching the definition of “urban area” in the reference, decreases with a larger UF threshold. Similarly, the False Positive Rate (FPR)—or commission error—decreases for increasing thresholds. This happens because less dense and smaller agglomerates of buildings, not considered as “urban area” in the reference, are increasingly discarded as the threshold increases. It is interesting to note that the most accurate results are obtained when spectral information is added, and “holes” in the UF extraction are filled.

**Figure 6.**
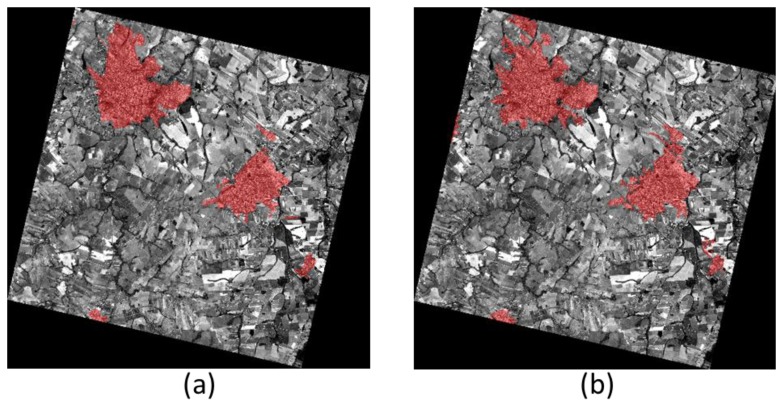
Aracatuba test site: (**a**) “compact urban area” reference data set superimposed to the original CBERS-2B scene; (**b**) results of the proposed procedure.

Adding a threshold on the minimum number of building hints per candidate urban area, the blue and green curves in Figure 5 are obtained. They show that the use of a rough estimate of the number of built-up structures improves the detection rate by reducing the FPR. The TPR value does not decrease as in the previous case, because the approximated number of structures simply discards smaller candidate areas that do not match the “urban area” model. Specifically, the graph shows that areas containing 50–100 buildings or more represent, at best, those captured by the available reference. For a visual comparison with the map in Figure 3a, the final extracted urban area extents are presented in Figure 3c. The overall accuracy is larger than 95%, with a commission error smaller than 2%. Similarly, for the surroundings of Aracatuba (depicted in Figure 6a), the overall accuracy of the results shown in Figure 6b against the same reference data set is 98%, with a commission error around 1.5%.

Please note that Figure 5 includes (orange squares) the results obtained applying the same procedure to five other CBERS scenes, showing similar (actually better) results.

To clarify the fusion steps for this test, please note that here the fused items are the candidate urban area extents identified by UF, the NDVI, and the number of building hints within each candidate urban area. As described in Section 3.3, the combination procedure is performed through AND/OR operations. In this case, the first operation is a logical OR between the candidate urban extents and an NDVI-based map of the natural elements of the landscape, obtained by means of user-defined thresholds (to include vegetation and/or water ponds). Then, the second operation is a logical AND between the output of the previous OR operation and the number of buildings within each candidate area.

### 4.3. Extraction of “Built-up Areas”

Since the second reference data set available for the Sao Paulo state corresponds to a definition of “urban areas” as agglomerates of built-up structures, in this second test we show that it is possible to use the same processing framework and tune it to a different urban area model. Specifically, in this case, no limitation on the number of buildings is necessary. Therefore, the output of the built-up structure detection at the finest spatial scale is not necessary. Instead, the original detection by the UF tool can be used, with low-pass filtering but without hole-filling, to avoid including urban parks and non-built-up areas. Additionally, and in order to discard isolated trees and pools, the spectral information is used to mask out points with very large and very small NDVI values. In this test case, therefore, the fusion is performed only by means of a logical AND between the candidate urban extents obtained by UF and the NDVI-based map (set to exclude vegetation and water areas wrongly classified by the UF step).

The corresponding final urban extents extraction results for Ribeirao Preto are presented in Figure 3d. Even from a preliminary visual inspection, it is clear that they differ significantly from those in Figure 3c. However, with respect to the second reference data set (Figure 3b) the overall accuracy of this map is greater than 95%, with a commission error below 3.5%.

### 4.4. Extraction of “Informal Settlements”

For the African test site, the “urban area” extraction problem refers to informal settlement detection. Informal settlements in this area are characterized by an average building size considerably smaller than for the majority of residential blocks, and a more compact (*i.e.*, with narrower roads) spatial pattern. They are also characterized by lower average reflectance, because of the employed materials. The aim of this test is to show that the proposed framework allows tuning the processing steps to match these properties.

**Figure 7.**
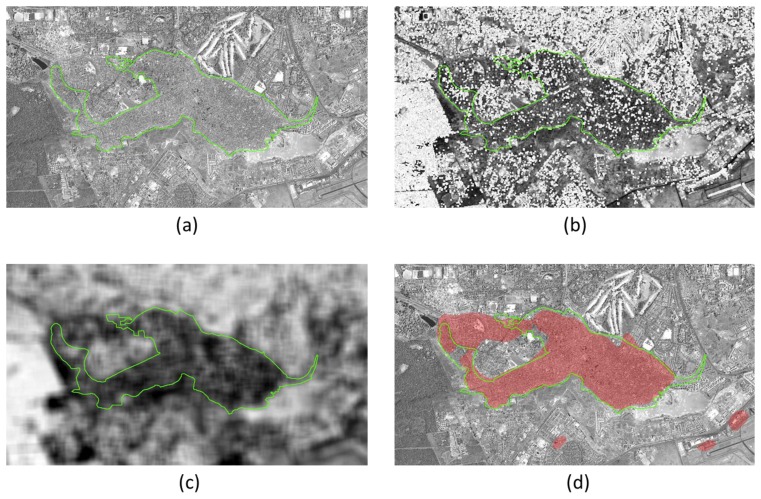
Results of the proposed procedure applied to the Kibera slum: (**a**) Quickbird panchromatic image, with superimposed the UN-Habitat layer; (**b**) output of the morphological opening filter; (**c**) output of the low-pass filter applied to the previous result; (**d**) final results, to be compared with the actual Kibera extents, in green.

Accordingly, the initial downsampling of the panchromatic image to 5-meter resolution was followed by a morphological opening filter instead of the UF (results are shown in Figure 7b). This option accounts for the different structure of the “urban area” to be detected, but still falls into the texture extraction step in Figure 1. Then, the usual low-pass filtering is used to remove false positives and to delineate more homogenous zones. Finally, a more accurate separation of informal settlements from rural regions with similar textural pattern is achieved by spectral information. Specifically, the average value of each band is computed, and lower values are selected to identify slums. In other words, in this test case the fusion inputs include candidate urban extents obtained by morphological filtering (as opposed to UF in the previous examples), and the average reflectance in the multispectral bands. In the end, the fusion is just a logical AND between the candidate areas and the average spectral values lower than a given threshold, to select high density settlement areas where buildings are mainly covered with low reflectance materials.

The results look promising for this test as well. Detected informal settlements, shown in Figure 4 on the right, can be compared with the UN-Habitat reference data on the left. However, a closer look to Figure 7d shows that the extracted extents for the well-known Kibera slum are neither very precise nor complete. The overall accuracy of the detection using the Quickbird dataset (see Figure 4) is 97.7%, with a commission error of 1.5%, but the Kappa coefficient is as low as 0.51. Indeed, it is important to stress that the difference between the extracted areas and the available ground truth are almost impossible to catch using EO data only, as the areas extracted by the algorithm match slum areas only in terms of spectral and spatial properties captured by the Quickbird sensor. As a matter of fact, the UN-Habitat definition of slums includes evaluation of sanitary, water, and economic features.

## 5. Discussion and Conclusions

A general comparison of all the quantitative evaluations performed for the three test sites is presented in Table 1. According to these figures, the methodology described in this paper was able to suite very different urban remote sensing tasks. The extracted urban areas range from large settlements with many houses, to mid-size to small built-up areas, to slums. Notably, these results are obtained by using the same multi-resolution spatial and spectral features, selected and combined according to very simple rules.

Indeed, as opposed to showing an approach that automatically detects urban areas and matches an available reference data, the aim of the previous section was to show that the proposed framework allows matching different definitions of “urban area” by acting on easily understandable parameters. The extraction of urban areas matching the first reference data in Sao Paulo, for instance, translates into “extraction of areas with high contrast with respect to their surroundings and with more than 100 bright objects”. Similarly, the extraction matching the second reference data set translates into “extraction of all areas with high contrast with respect to their surroundings, masking out vegetation and water bodies”. A more complex, but equally intuitive, definition refers to the example in Nairobi.

The important point we intend to stress here, therefore, is that the proposed approach provides a flexible framework that can adapt to multiple definitions. There is no “automatic” and “global” solution, but the technique has been applied with locally defined parameters in different parts of the world to recognize different kinds of urban areas. This is, in our opinion, a quite effective way to combine global remote sensing and local knowledge. In other words, on the one side one may exploit the global availability of EO data and the possibility to extract worldwide the same features with an objective and uniform methodology. On the other side, by “combining” these global features in a local way according to the proposed framework, one can extract many different sorts of “urban areas”.

In the same research direction, further improvements may be obtained at an intermediate spatial scale between “human settlements” and “built-up structures”, looking for a characterization of the image appearance of urban blocks by fusing linear elements (e.g., roads) to spectral and spatial features. Following this step, we hope that a more accurate characterization of all individual buildings, according to the land use class of their surrounding blocks, could become also available.

## Figures and Tables

**Table 1.** Mapping accuracy and commission errors for the three test sites.

**Test Case**	**Compact Urban Areas**		**Built-up Areas**		**Informal Settlements**	
		
	**Overall Accuracy**	**Commission Error**	**Overall Accuracy**	**Commission Error**	**Overall Accuracy**	**Commission Error**
Ribeirao Preto	96.8%	1.8%	96.9%	1.8%	-	-
Aracatuba	98.0%	1.5%	-	-	-	-
Nairobi	-	-	-	-	97.7%	1.46%
